# Free energy landscape and transition pathways from Watson–Crick to Hoogsteen base pairing in free duplex DNA

**DOI:** 10.1093/nar/gkv796

**Published:** 2015-08-06

**Authors:** Changwon Yang, Eunae Kim, Youngshang Pak

**Affiliations:** 1 Department of Chemistry and Institute of Functional Materials, Pusan National University, Busan 609-735, South Korea; 2 College of Pharmacy, Chosun University, Gwangju 501-759, South Korea

## Abstract

Houghton (HG) base pairing plays a central role in the DNA binding of proteins and small ligands. Probing detailed transition mechanism from Watson–Crick (WC) to HG base pair (bp) formation in duplex DNAs is of fundamental importance in terms of revealing intrinsic functions of double helical DNAs beyond their sequence determined functions. We investigated a free energy landscape of a free B-DNA with an adenosine–thymine (A–T) rich sequence to probe its conformational transition pathways from WC to HG base pairing. The free energy landscape was computed with a state-of-art two-dimensional umbrella molecular dynamics simulation at the all-atom level. The present simulation showed that in an isolated duplex DNA, the spontaneous transition from WC to HG bp takes place via multiple pathways. Notably, base flipping into the major and minor grooves was found to play an important role in forming these multiple transition pathways. This finding suggests that naked B-DNA under normal conditions has an inherent ability to form HG bps via spontaneous base opening events.

## INTRODUCTION

**Figure 1.**
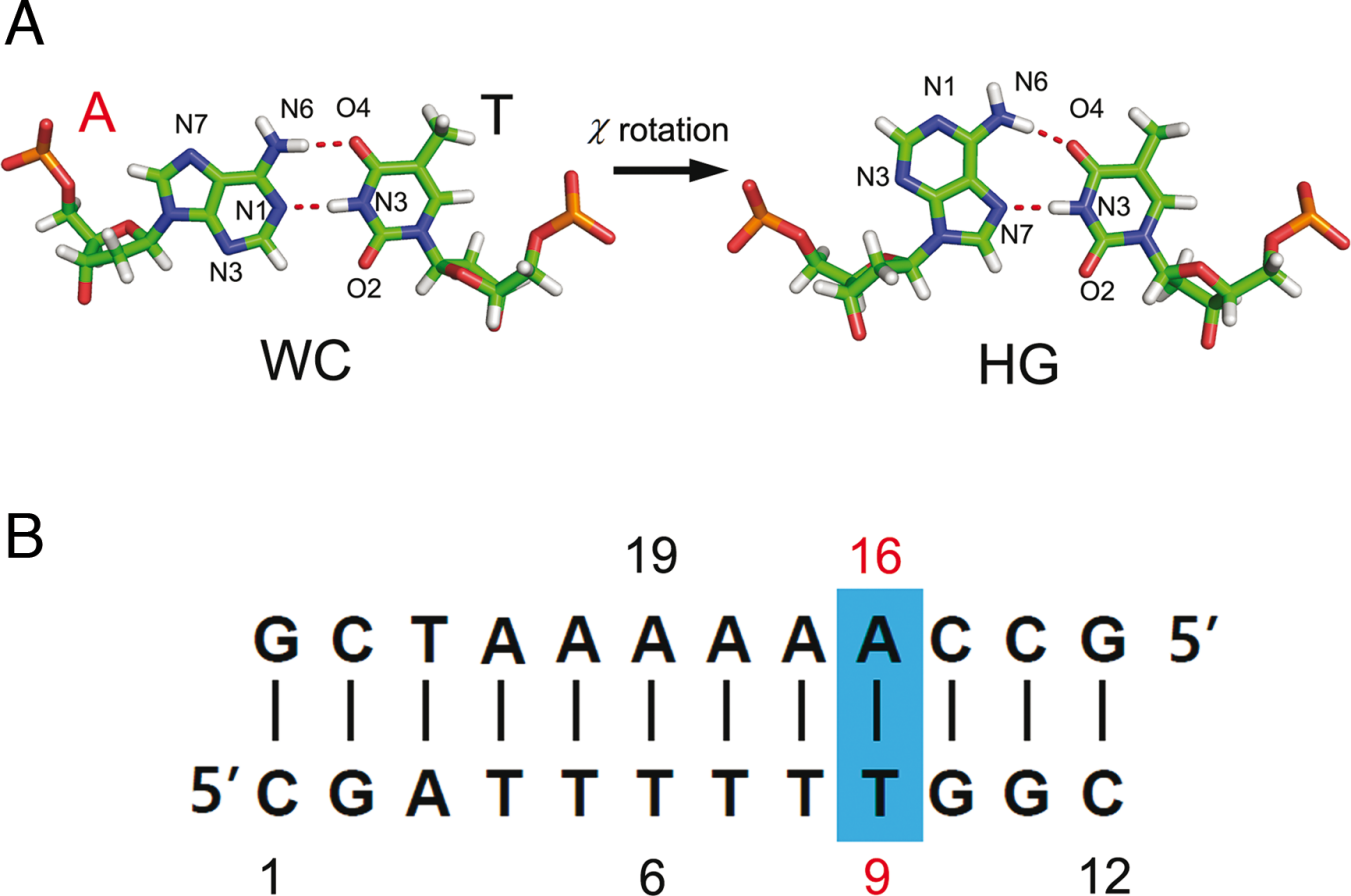
(**A**) Schematic diagram illustrating the transition from Watson–Crick (WC) to Hoogsteen (HG) base pairing. (**B**) Base sequence of the A_6_-DNA studied in this work. The A16-T9 base pair highlighted in a blue rectangular box is the location where the transition from the WC to HG base pair takes place. The adenine base (A16) is the target base for the base flipping and χ-angle rotation.

The inherent flexibilities of double helical DNAs facilitate spontaneous conformational fluctuations, which induce base pairs (bps) to adopt different geometries other than those dictated by the conventional Watson–Crick (WC) base pairing. Hoogsteen (HG) base pairing is an alternative base-pairing scheme for DNA double helices (Figure 1A) (1–3). Geometrically, HG bp in duplex DNAs can be formed by a 180° rotation of a purine target base (adenine A or guanine G) in a WC bp about its glycosidic bond (C1’-N9) from an *anti* (WC) to a *syn* (HG) conformation. Such excursion from the canonical base stacking places HG bps in substantially different structural and chemical environments and makes the HG bps specific binding sites for proteins and small drug molecules. The existence of HG DNA was first demonstrated in crystal by Subirana *et al*. (1). Until now, HG base pairing has been observed primarily in damaged DNAs and duplex DNAs complexed with enzymes and small ligands (4–10). However, for many reasons, experimental observation of HG bps in free duplex DNA has been a difficult and elusive task (11). Recently, a major breakthrough was made regarding the identification of such HG bps in naked B-DNA using a nuclear magnetic resonance (NMR) relaxation method. More specifically, Nikolova *et al*. were able to trap the HG bp in free adenine–thymine (A–T) rich DNA duplexes (12). Notably, they showed that this alternative bp can exist as transient entities in thermal equilibrium with the WC bp and that for example lifetimes of the transient HG bp state of A_6_-DNA are 0.3 ms for A–T pair and 1.5 ms for G–C pair (See Figure 1B for the sequence of A_6_-DNA). This finding is of great importance, because it shows that a free double helical DNA has an intrinsic ability to modulate its function by undergoing a spontaneous conformational switch between WC and HG bps. Furthermore, a more recent NMR study on an expanded test set of free duplex DNAs indicated that transient HG bps could be formed more broadly across diverse sequence and positional contexts (13). Although earlier NMR studies significantly advanced our understanding of the dynamics and thermodynamic stability of the HG bp state by measuring transition rates and equilibrium constants between the WC and HG bp states, detailed pathways for these bp transitions have not been resolved experimentally.

**Figure 2.**
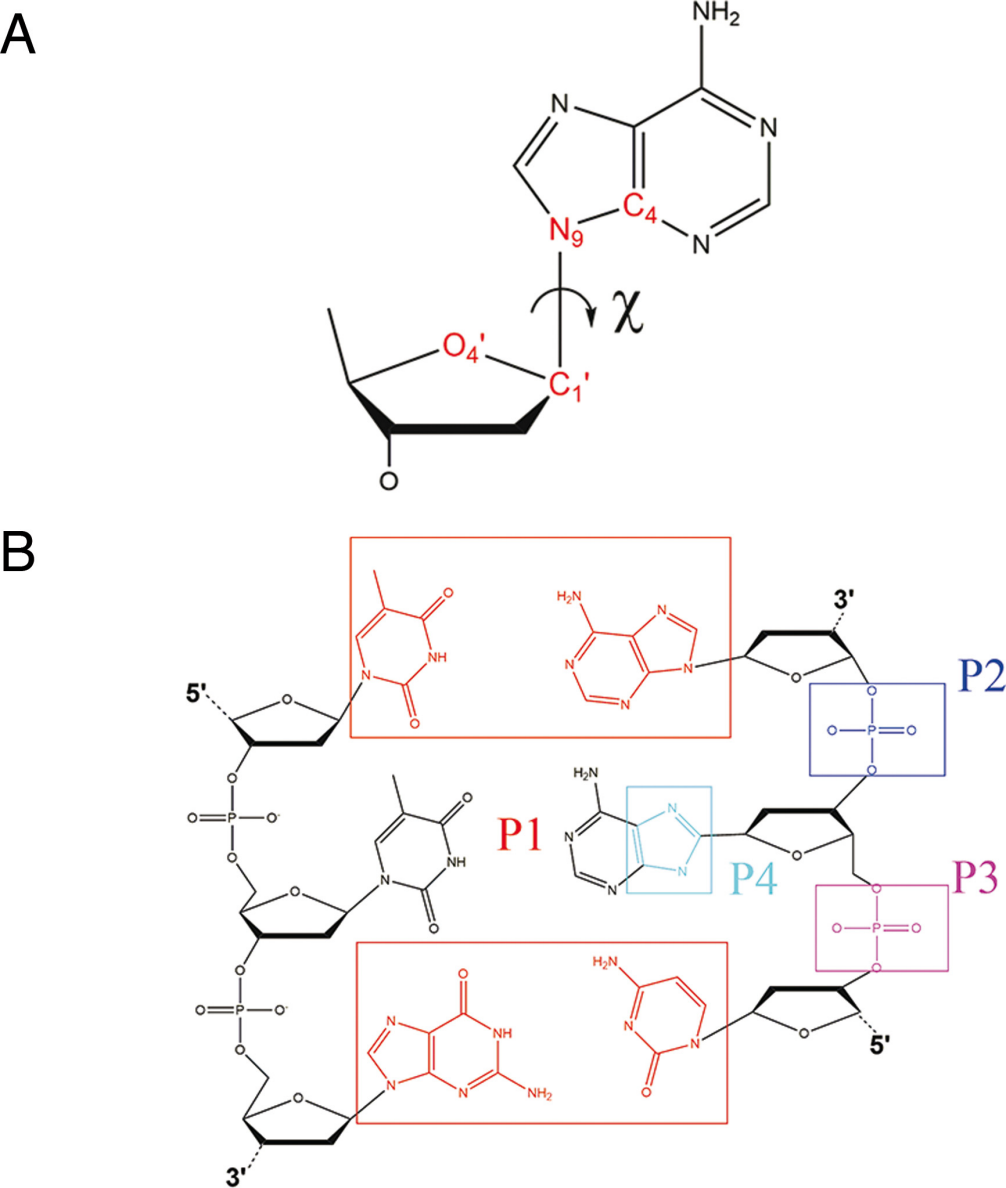
(**A**) Definition of glycosidic angle (χ-angle) for adenine (purine residue in middle of the right side). The χ-angle is a dihedral angle defined by four consecutive atoms: O4’, C1’, N9 and C4. The glycosidic bond is defined between C1’ and N9. An *anti-*conformation such as WC conformer is defined for -180° ≤ χ ≤ -90° and a *syn*-conformation such as HG conformer is defined for −90° ≤ χ ≤ 90°. (**B**) Definition of CPDb angle. This angle is a pseudodihedral angle consisting of P1-P2-P3-P4. The P1 is the center of mass of the atoms enclosed by the red rectangle, which corresponds to the upper and lower base pairs. The P2 and P3 are the center of masses for two phosphate groups. The P4 is the center of mass of the five-membered ring in the target purine base. Base flipping into the major and minor grooves corresponds to CPDb > 0° and CPDb < 0°, respectively.

All-atom molecular dynamics (MD) simulations provide a powerful computational means for investigating biomolecular systems, and can be used for studying conformational dynamics and structures of the WC and HG bp states in DNA duplexes. In earlier simulation studies, Orozco *et al*. (14) performed MD simulations on antiparallel HG duplexes (poly AT sequences) to probe their stability in aqueous environment. Their study indicated that the HG duplex closely captured the B-form DNA and could be a feasible structure in water. Recently, an all-atom level conjugate peak refinement (CPR) method (15) under generalized Born implicit solvation (16,17) for several A-tract duplexes suggested a possibility of multiple routes for the WC/HG bp switching based on the minimum potential energy criterion (12). Although this CPR result shed light on mechanistic aspects of bp transitions, it raised interpretative concerns due to the complete omission of an entropic contribution (-*TΔS*). Furthermore, this computational approach involved the use of the implicit solvation model, which was recently shown to overestimate overall energetics for base flipping of duplex DNAs (18). Therefore, to produce more reliable pictures of the bp transition process, an all-atom level computation of free energy landscape under explicit solvation is required. As shown by the previous NMR study (12), the bp switching is known to have large free energy barriers, and this constitutes a computational bottleneck for exploring conformational space relevant to such transitions. Therefore, for the production of free energy landscapes involving substantial activation energy barriers, the umbrella sampling MD simulation offers a promising computational strategy. This method can yield a potential of mean force (PMF), which corresponds to a free energy profile as a function of reaction coordinates. In the context of the umbrella sampling MD, PMF profiles can be simulated by introducing a set of harmonic biasing potentials centered at discrete points of predefined reaction coordinates. In this regard, proper reaction coordinates should be defined for PMF simulations. In the transition from WC to HG base pairing for the target A–T bp, the χ-angle of the purine base is a natural variable describing the glycosidic bond rotation from an *anti* to a *syn* position, thereby making this angle one of the possible reaction coordinates (Figure 2A). Furthermore, it is likely that the χ-angle rotation can occur in concert with base flipping. Bp flipping (or base opening) is an excursion of a target base in the canonical stacking (A–T or G–C) to an extrahelical position and is coupled to changes in specific backbone dihedrals and certain helical distortions such as untwisting and bending. This base flipping process allows DNA modifying or repair enzymes to access specific bases in duplex DNAs and is recognized as a mechanistic requirement for translation, DNA replication and DNA repair. Due to its fundamental importance for understanding the intrinsic functions of duplex DNAs, base flipping has been the subject of extensive experiments (19,20) and computer simulations (18,21–26). In particular, previous all-atom base flipping simulations on free DNA (18,21–26) and protein/DNA complex (27–29) have provided much insight of this event at atomistic resolution. All these simulations emphasized purely base opening, indicating that purine base flipping has a lower free energy barrier than that of pyrimidine case and occurs more favorably in the major groove than in the minor groove. Base flipping is energetically unfavorable due to the disruption of hydrogen bonds in the WC state and loss of stacking interactions with neighboring bps. Nevertheless, as shown by previous NMR studies on imino proton exchange (30), base flipping occurs spontaneously, which implies that base flipping is an essential mode of conformational fluctuations of duplex DNAs and that it may well be operative in the transition between WC and HG bps. In view of base stacking of B-DNA, *anti–syn* rotation of the χ-angle of the target base can be hindered by repulsive interactions with neighboring bases. In this regard, base flipping appears to beneficially promote χ-angle rotation by effectively reducing the energetic cost associated with the hindered rotation. As a consequence, in the present description of PMF, base flipping and χ-angle rotation were taken into account, though this demanded a more challenging PMF computation in two dimensions. In contrast with the geometrically well-defined χ-angle, no unique variable has been defined as a reaction coordinate of base flipping. Until now, several variants of pseudodihedral angles have been proposed to describe this process. As was previously shown by one-dimensional (1D) PMF simulation (26), a pseudodihedral angle (CPDb) appears to be an effective variable for simulating the base flipping process (Figure 2B). Therefore, both CPDb and χ-angles need to be included as a set of two-dimensional (2D) reaction coordinates for the present PMF simulation. However, no 2D-PMF profile for such transition has been reported probably because 2D-PMF simulations are computationally challenging due to MD sampling of a large number of umbrella windows covering a wide range of 2D conformational space (CPDb, χ).

In the present study, we call our attention to A_6_-DNA which has been fully investigated with the earlier NMR and computer simulation studies (12). The aforementioned CPDb and χ-angles were employed as reaction coordinates for the adenine in the target bp of A16:T9 in A_6_-DNA (Figure 1B), and then 2D-umbrella MD simulation was performed using a modified version of the amber99bsc0 force field (31) and the tip3p water model (4). A reasonably well-converged 2D-PMF profile for the WC/HG switching was obtained by reweighting a set of biased ensembles sampled at 300 K. This reweighting procedure was carried out using the variational free energy profile (vFEP) method (32,33). The vFEP scheme appears to be very effective in the construction of free energy profiles by dealing with multidimensional umbrella sampling data. In particular, this method was shown to produce robust PMF profiles even with smaller numbers of data points and umbrella windows. Based on the simulated 2D-PMF profile, detailed features of transition pathways joining WC and HG bp states were fully characterized.

The simulated 2D-free energy surface at 300 K shows that the transition between the WC and HG bp states proceeds via multiple routes with a range of activation free energy barriers (10–14 kcal/mol). Interestingly, all these pathways displayed various degrees of base flipping, which suggests that spontaneous base flipping plays a role in the bp transition.

## MATERIALS AND METHODS

### Force field

The choice of the force field is important especially for DNA simulations. Although the amber99bsc0 force field (31) was one possible choice for B-DNA simulations, herein a modified version of amber99bsc0 was employed. The amber99bsc0 was derived from the standard amber99 force field (34) to improve structural features of B-DNAs by adding α (O3’-P-O5’-C5’) and γ (O5’-C5’-C4’-C3’) torsional terms for the sugar-backbone parameters. It is well known that amber99bsc0 preserves B-DNA. However, Chen *et al*. (35) demonstrated that amber99 somewhat overestimates the strength of base stacking in nucleic acids and biases the χ-angle energy profile. In an effort to improve this force field, they optimized a set of parameters specific for purine and pyrimidine bases. Their correction scheme includes the modification of the Lennard–Jones (L–J) parameters of the heavy atoms (C, N, O) of bases in nucleic acids and the OW atom of water. Furthermore, in the modification scheme mentioned above, the χ-torsional term was adjusted to reproduce experimental *anti–syn* populations of ribonucleosides and deoxyribonucleosides. Accordingly, these modified parameters were substituted for the relevant parameters of amber99bsc0 and used in the present PMF simulation.

### Aggregation simulations of deoxyribonucleosides in water

In an effort to justify a modified version of the amber99bsc0 force field mentioned above, association constants *K_A_* of deoxyadenosines (dAs) and deoxythymidines (dTs) were calculated from 50 ns aggregation simulations at constant temperature (298 K) and pressure (1 atm) using this force field in conjunction with the tip3p water. The temperature and pressure were controlled by the modified Berendsen thermostat (36) and Berendsen barostat (37), respectively. For the aggregation simulation of dAs in water, a total number of 16 dAs were solvated by 19 731, 23 677 and 29 596 tip3p water molecules for the three concentrations of 0.0450, 0.0375, 0.0300 *m* (moles/kg solvent), respectively. From three independent simulations for these concentrations, the cluster analysis was performed using one of the gromacs tools (g_clustsize) (38) with respect to the size of aggregation (monomers, dimers, etc.) and then the association constant *K_A_* was obtained by the least-square fitting (39). For the aggregation simulation of dTs in water, a total number of 16 dTs were solvated by 4439, 5919 and 8879 tip3p water molecules for three concentrations of 0.20, 0.15 and 0.10 *m*. The aggregation state is defined if the distance of centers of mass of any two molecules is within 3.5 Å. The osmostic coefficient *ϕ* (40) is defined as

(1) \begin{equation*} \phi = \frac{{m_N }}{{m_T }} = \frac{{\sum\limits_i {[A_i ]} }}{{\sum\limits_i {i \cdot [A_i ]} }}, \end{equation*}

where [*A_i_*] is equilibrium molal concentration of *i*-mer species, *m_N_* is sum of equilibrium molal concentration of monomers, dimers, trimers, etc. and *m_T_* is initial molal concentration of monomer. From the equal *K* model (assuming all association constants for dimerization, trimerization, etc. are the same) (39), the association constant *K_A_* can be obtained from

(2) \begin{equation*} \frac{{(1 - \phi )}}{{\phi ^2 }} = K_A \cdot m_T . \end{equation*}

The resulting association constants computed from the MD trajectories using the original amber99bsc0 and modified amber99bsc0 force fields are given in Supplementary Figure S1.

### Simulation system for base pair transition

The A_6_-DNA is a target system for studying the free energy landscape of bp transitions (Figure 1B). This DNA is an A–T rich double helix with a B-DNA structure, upon which an NMR relaxation study was previously conducted (12). The present simulation system consists of a single A_6_-DNA molecule solvated with 8322 tip3p water molecules in a cubic simulation box of an initial size 56.9 Å. To insure the compliance with the charge neutrality condition, a total number of 22 sodium ions were inserted into the system. L–J potential energies were computed using a cutoff distance of 10 Å. Electrostatic potential energies were calculated using the particle-mesh Ewald method (41,42) and a cutoff distance of 10 Å. All bond distances between heavy and hydrogen atoms were fixed by the LINCS algorithm (43). A time step of 2.0 fs was used. Initially, this system was equilibrated for 5.0 ns at 300 K and 1.0 atm. The coupling constants for the pressure and temperature control were 1.0 and 0.1 ps, respectively. After a relaxation period of 5.0 ns, the final box size becomes 55.8 Å, which was then fixed for the umbrella sampling MD.

### Potential of mean force calculations

PMF was defined in two dimensions spanned by (CPDb, χ). A total number of 13 × 24 umbrella windows was set up, such that -100° ≤ CPDb ≤ 100° and -180° ≤ χ ≤ 180°. For the CPDb angle, umbrella samplings of the entire CPDb space are not pursued here, since free energy values beyond this sampling interval are too large to produce energetically realistic pathways. In fact, the present range of CPDb angles seems sufficient, since the basis is virtually flipped out at CPDb > |60°|. The umbrella potential at a rectangular grid point (*i*, *j*) is defined as

(3) \begin{eqnarray*} &&U_{i,j} (CPDb,\chi ) \\ \nonumber &=& k_{CPDb} \cdot (CPDb - CPDb_i^0 )^2 + k_\chi \cdot (\chi - \chi _j^0 )^2, \end{eqnarray*}

where *k*_CPDb_ and *k_χ_* are the umbrella harmonic force constants for the CPDb and glycosidic angles, respectively. The $CPDb_i^0$ and $\chi _j^0$ are the window center values at a grid point (*i*, *j*). Here, the *k*_CPDb_ and *k_χ_* values were chosen to be 100 and 50 kcal/mol, respectively.

Setting up initial structures for the umbrella MD sampling is not a trivial task, since the umbrella windows need to cover an extensive range of the conformational space for CPDb and glycosidic angles. Instead of modeling one initial structure at a time for each window and repeating this process sequentially up to 13 × 24 times, we employed a well-tempered metadynamics scheme (44) to generate all relevant initial structures for the present umbrella MD runs. This is an enhanced MD method for effective conformational search. For the two reaction coordinates of CPDb and χ-angles of the A16 base, history-dependent external biasing potential (Gaussian type potential) is deposited along the metadynamics trajectory for each reaction coordinate. For the metadynamics setting, the initial hill size was 1.0 kcal/mol and the hill deposition was applied every 1.0 ps. In order to control the biasing potential to a manageable level, a bias factor of *γ* = 15 was used. The bias factor (44) is defined as

(4) \begin{equation*} \gamma = \frac{{T + \Delta T}}{T}, \end{equation*}

where *T* and (*T* + Δ*T*) are simulation temperature of system and fictitious temperature of collective variables (CPDb and glycosidic angles), respectively. Although this metadynamics MD can sample a broad conformational space spanned by CPDb and χ-angles in a relatively short time, severe breakdown of the B-DNA backbone structure was frequently observed in the trajectory. Thus, another measure was taken to circumvent this unrealistic disruption of the DNA backbone. Here, the heavy atom positions of the upper (A17-T8) and lower (C15-G10) bps from the target A16-T9 pair position were restrained with a force constant of 2.4 kcal/Å^2^, which well maintains the B-DNA backbone during the exploration of high energy conformations. The resulting metadynamics trajectory for 50 ns is shown in Supplementary Figure S2. Note that almost every window region is visited by the trajectory. Thus, any conformation falling into a window could be a possible initial structure for that window. Furthermore, when a window region is missed by the trajectory, any conformation in nearby windows can be chosen as an alternative initial structure. For each umbrella window, an initial structure prepared in this way was equilibrated for 1.0 ns under the corresponding umbrella potential without the heavy atom restraint of the neighboring bps.

For the umbrella MD simulation, MD at a canonical ensemble was applied using the modified Berendsen thermostat at 300 K (36). For each umbrella window, starting from the initial structure described above, the umbrella MD simulation was carried out for 20 ns. All biased MD trajectories were combined together to construct the 2D-PMF profile using the vFEP method, which is an effective reweighting scheme based on maximum likelihood functions in conjunction with direct spline interpolations of free energy profiles (32,33). It has been demonstrated that vFEP method can produce robust and reliable free energy profiles even with relatively small numbers of windows and data points in 1D and 2D grids. All simulations were carried out using Gromacs program (v 4.5.3) (45) and Plumed program (v 1.3.0) (46).

### Simulation convergence

The free energy surface of the bp transition was constructed by using the vFEP program (32,33) from a total number of 312 biased MD ensembles each of simulation length 20 ns. In order to check the convergence of this free energy map, we constructed cumulative free energy surfaces with respect to simulation time. A series of free energy maps for cumulative times of 5, 10, 15 and 20 ns are provided in Supplementary Figure S3a, and the errors relative to the free energy map for the cumulative time of 20 ns are shown in Supplementary Figure S3b. The time series of the free energy surface appeared to remain virtually the same after 10 ns, and the root mean square error of this energy surface was less than *k_B_*T.

### Structural analysis of duplex DNA

To analyze the overall conformations of nucleic acids, we used the 3DNA program (47,48). This program produces a set of very extensive DNA bp modes: the intra-bp modes (shear, stretch, stagger, buckle, propeller, opening) and the inter-bp modes (shift, slide, rise, tilt, roll, twist) (49). These bp modes provide comprehensive descriptions of the overall deformation of duplex DNA. For two force fields (amber99bsc0 and modified amber99bsc0), DNA bp parameters were compared.

## RESULTS

### Force field validation

For the simulation of duplex DNAs, the amber99bsc0 force field (31), a variant of amber99 force field (34), would be a suitable choice due to its better description of B-DNA structures. In this study, we attempted to further improve amber99bsc0 by employing a modified set of parameters specifically optimized for base stacking and glycosidic torsion in ribonucleic acids (RNA) (35). Since changes in these parameters were limited to only the RNA bases, we proposed to apply these parameters to the bases of duplex DNA. In an effort to justify this correction scheme for DNA bases, we investigated two association constants of deoxyguanosines and dTs in water at ambient temperature. In a similar manner to the case of ribonucleosides, the amber99bsc0 enhanced aggregation of deoxyribonucleosides in water, but the inclusion of the correction scheme into amber99bsc0 produced association constants more conforming to the experimental result (Supplementary Figure S1). Thus, the base stacking correction scheme by Chen *et al*. (35) seems well transferable to DNA studies without further optimization.

**Figure 3.**
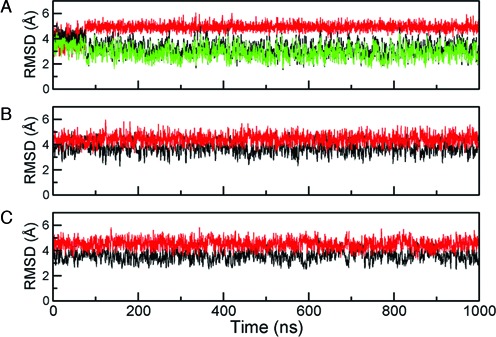
Time series of the root mean square deviation (RMSD) of (**A**) Dickerson dodecamer (DD), (**B**) WC and (**C**) HG base pairs of A_6_-DNA. Three independent MD simulations at 300 K and 1 atm were performed for 1 μs. All-atom RMSD values excluding terminal base pairs were computed relative to the references on an ideal canonical B-form (black) and an ideal canonical A-form (red). For DD, another RMSD profile calculated from nuclear magnetic resonance driven B-form (PDB entry: 1NAJ) is colored green and its averaged RMSD value is 2.8 (0.7) Å.

For additional tests of the present force field, three independent MD simulations on the WC and HG bp states of A_6_-DNA and the B-form of Dickerson dodecamer (DD) (PDB entry: 1NAJ) (50) were performed for 1 μs at 300 K. From these trajectories, the time series of all-atom root mean square deviation (RMSD) values of duplex DNA are given in Figure 3. The averaged RMSD values of DD and the WC and HG of A_6_-DNA with reference to the corresponding ideal B-form DNAs are about 3.0 (0.6) Å, 3.7 (0.5) Å and 3.6 (0.6) Å, respectively. In addition, when the same profiles were calculated from ideal A-form references, the RMSD values are about 4.9 (0.3) Å, 4.4 (0.7) Å, 4.5 (0.7) Å, respectively. Apparently, this result indicates that all three DNA systems prefer B-DNA. One notable feature is that the B-form referenced RMSD value of A_6_-DNA is larger than that of DD, suggesting that A_6_-DNA deviates more from the ideal B-form than DD does. In an effort to trace such extra distortions of the A-tract oligomer relative to DD, various DNA structural parameters were analyzed using the 3DNA program (47,48). Distributions of local DNA parameters, pseudorotation angle (C1’-C2’-C3’-C4’) in sugar ring (51) and delta torsional angle (C5’-C4’-C3’-O3’) are shown in Supplementary Figure S4. Regarding the local DNA parameters for both DD and A_6_-DNA (Supplementary Figure S4a), the current force field produced some notable deviations of slide and twist relative to the survey data and the amber99bsc0 result. Obviously, such deviations could be an artifact of the modified force field. Despite caveats in that force field, the predicted ranges of pseudorotation and delta torsional angles of A_6_-DNA and DD (Supplementary Figure S4b) agree with those of B-form duplex (52). For other base parameters computed, A_6_-DNA reveals various degrees of deviations from those of DD. More specifically, A_6_-DNA displays increased magnitudes of roll, tilt, inclination and propeller twist. To provide more insight into structural features of A-tract, such parameter values were recomputed for each bp position (Supplementary Figure S5). For A_6_-DNA and DD computed, their tilt parameters behave similarly, virtually staying around 0° along the sequence (Supplementary Figure S5a). Regarding the simulated roll parameter, for DD the roll value is steadily fluctuating around −2.5°. On the other hand, for A_6_-DNA this parameter remains close to zero mainly in the A-tract, but noticeable change is observed near the junction between the A-tract and GC flank (Supplementary Figure S5b). This means that A_6_-DNA would be bent primarily at that junction region. This observation is in line with the junction model previously suggested for explaining bending of A-tract (53). In earlier investigations (49–52), it was reported that enhanced propeller twists in A-tract induce the A-tract to be more rigid and straight by generating bifurcated hydrogen bonds. In our simulation, compared to DD, somewhat increased propeller twist for A_6_-DNA was found in the A-tract (Supplementary Figure S5c). Therefore, the present force field seems to capture some structural features of A-tract at qualitative level.

### Multiple transition pathways between WC and HG

**Figure 4.**
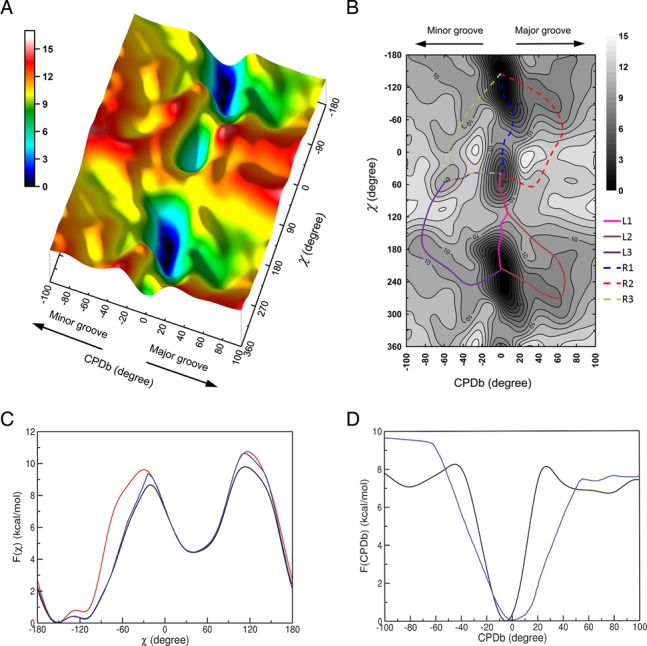
(**A**) Free energy landscape as a function of CPDb and χ-angles (in degrees) at 300 K. (**B**) 2D-free energy map with possible transition pathways denoted. The six transition pathways are named as R1, R2 and R3 for clockwise rotation of χ and L1, L2 and L3 for counterclockwise rotation of χ. 1D-free energy profiles along (**C**) χ and (**D**) CPDb. In the panels of (C) and (D), the black lines represent effective 1D-PMFs, which were obtained by using $F(\chi ) = - RT \cdot \ln \left[ {\int {P(\chi ,CPDb)dCPDb} } \right]$ and $F(CPDb) = - RT \cdot \ln \left[ {\int {P(\chi ,CPDb)d\chi } } \right]$, where *P*(*χ*,CPDb) is the simulated 2D probability distribution function. The red line in the panel (C) represents 1D-PMF profile of χ-angle in the absence of base flipping (CPDb = 0°). The blue lines in the panels of (C) and (D) describe the lowest 1D-free energy paths with respect to χ and CPDb, respectively.

The free energy surface for the transition from WC to HG base pairing at 300 K is given in Figure 4. The topography of the free energy surface was not symmetric with respect to the base flipping via major and minor grooves. The free energy cost to break up the WC bp by the base flipping (up to CPDb = ±60°) was somewhat higher in the minor groove than in the major groove. However, once the target base fully escapes from the WC basin, the free energy profile is rather flat and there appears to be little energetic difference between the major and minor groove pathways. The free energy difference between the WC and HG pair states was 4.4 kcal/mol, which is somewhat larger than the NMR result (3.0 kcal/mol) (12). Besides the WC and HG pair states, it is notable that a locally stable intermediate state (LS) [(CPDb, χ) = (-57.3, 51.2)] was present in the minor groove, whereas no meaningful intermediate state was observed in the major groove. The stability of this intermediate state in the minor groove is ascribed to enhance stacking interactions between locally deformed bases in the flipped-out state. An inspection of the free energy surface revealed at least six transition pathways (R1, R2, R3; L1, L2, L3) joining the WC and HG pair states. The existence of multiple pathways is due to base flipping into either the major or minor groove in combination with possible clockwise and counterclockwise rotations of χ-angle. In the clockwise rotation, the A16 purine base is driven to make an edge to face contact with the C17 pyrimidine base, while in the counterclockwise rotation, the A16 encounters a similar contact with the A15 purine base. Clockwise χ-angle rotation produced a group of pathways R1, R2 and R3, whereas counterclockwise rotation yielded another group of pathways L1, L2 and L3. As shown in Figure 4A and B, the pathways R1 and L1 belong to a group of small base flipping pathways (CPDb ≤ |20°|). In this group of pathways, the transition from the WC to HG bp state is rather straightforward and initiated by χ-angle rotation without much change in CPDb angle. Furthermore, it proceeds toward the HG bp formation through the TS by allowing only a slight degree of base flipping into the major groove. The other group consisting of the four pathways (R2, R3, L2 and L3) involves rather large base flipping processes (CPDb ≥ |60°|) that deviate substantially from the small base flipping pathways. In this case, the base flipping is accompanied by the χ-angle rotation and the substantial base flipping drives the target base virtually into a position on the outside of the double helix. Subsequently, from this opened state, the target base gradually returns to the closed state for the HG base pairing. The major groove opening pathways (R2 and L2) exhibit a two-state like behavior, whereas the minor groove opening pathways (R3 and L3) seem to deviate from the two-state model due to possible trapping in the local intermediate state en route.

**Figure 5.**
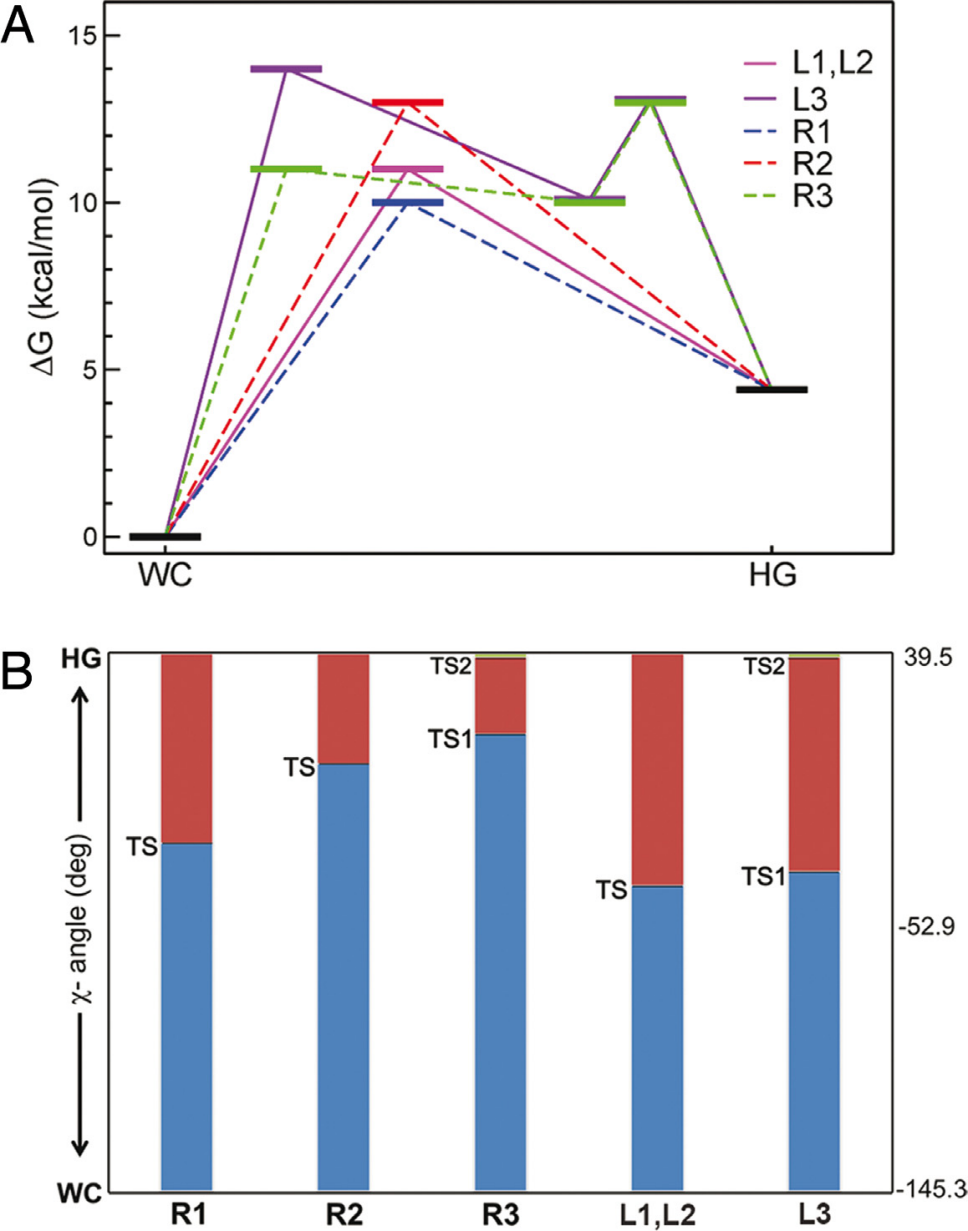
(**A**) Schematic diagram of the free energy levels of several conformational states (WC, LS, TS and HG) along each of the six transition pathways. (**B**) Comparison of glycosidic angles in degrees corresponding to various transition states along each pathway. All transition states fall into *syn*-state.

The free energy map reveals a total of six TSs located in the major and minor grooves. The major groove opening pathways exhibits a single TS, whereas the minor groove opening pathways are associated with two TSs (TS1 and TS2). The forward activation energy barriers from the WC to HG base pairing are distributed in the ranges of 10–14 kcal/mol. As shown in Figure 4B, the small base opening pathways (R1, L1; 10–11 kcal/mol of barrier) are energetically more favorable than the large base opening pathways (R2, R3, L2, L3; 13–14 kcal/mol of barrier). Furthermore, as shown in 1D-PMF profile of the glycosidic angle (χ) rotation (Figure 4C), the activation energy barrier is somewhat higher in the absence of base flipping (CPDb = 0°) than in the presence of base flipping. This implies that base flipping could play a role in the energetics of bp transition. On the other hand, as indicated in 1D-PMF of CPDb (Figure 4D), the major and minor groove base opening processes involve similar magnitudes of activation free energy barrier: 8.0 and 8.4 kcal/mol via the major and minor groove pathways, respectively. These barriers are in line with a purine opening simulation result of Lavery *et al*. (23) using amber99 (7–9 kcal/mol). In summary, a schematic diagram of individual energetics of the transition pathways is given in Figure 5A and the progress of *anti–syn* transition for TS in terms of χ-angle is given in Figure 5B. This figure demonstrates that all TSs are in the *syn*-state, indicative of the TS being physically closer to the HG bp state (*syn*) in terms of the χ-angle. This finding is in agreement with a previous interpretation of a late TS based on the Φ-value analysis of the NMR data (13).

**Figure 6.**
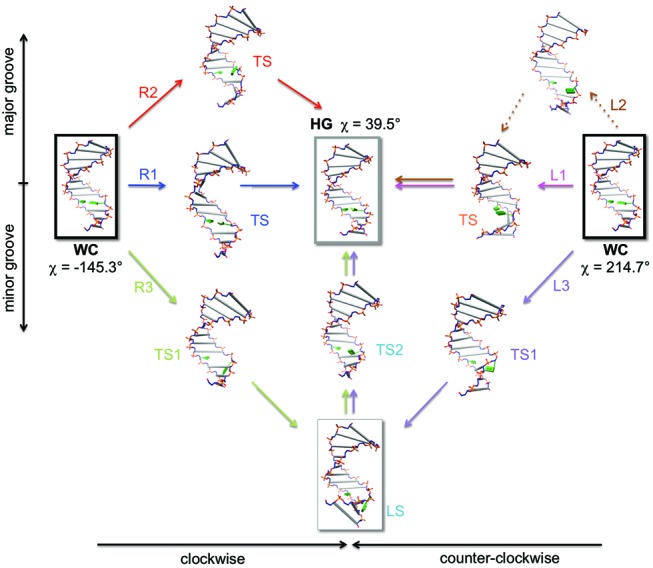
Representative snapshots along each transition pathway. Here, transition states (TS, TS1 and TS2) and local intermediate state (LS) are shown. The target base pair (A16-T9) of A_6_-DNA is modeled as green rectangular boxes and other base pairs are represented as gray rods. Note that the large base opening pathways via the minor groove (R3 and L3) share the later stage of the transition. Also two counterclockwise transition pathways via the major groove (L1 and L2) were merged after passing their common TS.

Representative snapshots of simulated pathways are shown in Figure 6. This result indicates that deformations of the double helix occur in complex ways, but the structural integrity of B-DNA is reasonably maintained during the course of transition. Although the discrete snapshots illustrated in this figure give useful molecular pictures of the transition process, detailed distortions of the duplex DNA caused by the local bp transition may not be fully captured in Figure 6. In an attempt to provide more comprehensive descriptions of these pathways, a number of conformational snapshots for each pathway were processed for animation using the Chimera program (54). The corresponding movie files for all six transition pathways are provided in Supplementary data. It is worthwhile to note that the overall structural responses of duplex DNA to local bp transition are dramatically flexible and elastic, as depicted by the movie files.

## DISCUSSION

Detailed transition mechanism between the WC and HG bp in a naked DNA can provide a molecular basis for elucidating inherent functions of duplex DNAs beyond their genetic codes. To gain insight of transition pathways from WC to HG base pairing in duplex DNAs, a reasonably converged free energy profile of the A-tract was obtained from the PMF simulation of 20 ns as a function of the 2D reaction coordinates. Considering that the flexibility of duplex DNA causes spontaneous structural fluctuations through base flipping (11), the inclusions of CPDb angle for base flipping and χ-angle for *anti–syn* rotation were necessary to obtain realistic descriptions of free energy profiles. Our simulated 2D-PMF profile at ambient temperature showed that in addition to the free energy minimum basins of WC and HG bp states, a relatively stable intermediate state is located in the minor groove pathway. The current free energy landscape demonstrated the existence of multiple transition routes connecting the WC and HG bp states. Hence, various TSs corresponding to such multiple pathways were located and their forward activation free barriers are distributed in a relatively narrow range of 10–14 kcal/mol. Unfortunately, the magnitude of simulated TS barriers was somewhat smaller than the NMR value (16 kcal/mol). This underestimation may be due to adverse effects of using the modified L–J parameters (35). As shown in this study, this modification clearly improves base stacking behaviors and χ-angle profiles, but somewhat deteriorates accuracy of some DNA bp parameters. Noting that such simple corrections could possibly unbalance the force field, a care needs to be taken when this L–J correction is applied. Eventually, the gap between simulation and experiment should be closed through further improvement of the force field.

It should be noted that all these six predicted pathways involve various degrees of base flipping toward the major and minor grooves. Furthermore, clockwise or counterclockwise rotations of the χ-angle of the purine base added extra diversity to the shaping of the multiple transition pathways. Depending on the extent of base flipping, overall pathways were divided into two groups of pathways, that is, small and large base opening routes. The small base opening pathways are shortcuts in which the bp transition occurs inside the DNA duplex, whereas the large base opening pathways are detours involving substantial distortion of the target base out of the double helix. Energetically, the bp transition appears to be more likely to occur via small base opening (R1 and L1).

In the current simulation, we showed that spontaneous fluctuation of free B-DNA in solution could lead to formation of the HG bp state via diverse transition routes. The existence of such multiple pathways between WC and HG bp were also suggested in a previous CPR simulation using the CHARMM27 force field (12). In the CPR study, however, a possibility of the minor groove pathway was completely ruled out. In contrast, the present simulation approach in the context of 2D-PMF provides more balanced and extended pictures of HG breathing via major and minor grooves. Notably, taking advantage of our 2D-free energy surface, effective 1D-PMFs in terms of χ-angle show that the base flipping mode plays a role in reducing the activation free energy barriers of bp transition by some extent. Clearly, such HG breathing is manifested in intrinsic flexibility of free B-DNA. Although the present simulation was focused on a free B-DNA in solution, its application to DNA/protein complexes would be more interesting. In the case of DNA/protein complexes, protein binding can enhance the base opening process by greatly reducing free energy barriers through specific and favorable protein–DNA interactions (28). Therefore, one would expect that upon binding to proteins, transition pathways between the WC and HG base pairing would be altered and that the large base opening pathways (R2, R3, L2, L3) might possibly be facilitated by DNA–protein interactions.

## Supplementary Material

SUPPLEMENTARY DATA
