# Supplementary material for: Free energy landscape and transition pathways from Watson–Crick to Hoogsteen base pairing in free duplex DNA
Source: Nucleic Acids Res. 2015 Aug 6;43(16):7769–78. doi: 10.1093/nar/gkv796 (PMC4652778; doi:10.1093/nar/gkv796)
Supplement: SUPPLEMENTARY DATA [file supp_43_16_7769__index.html]

Free energy landscape and transition pathways from Watson–Crick to Hoogsteen base pairing in free duplex DNA — SUPPLEMENTARY DATA 

# Free energy landscape and transition pathways from Watson–Crick to Hoogsteen base pairing in free duplex DNA

## SUPPLEMENTARY DATA

- SUPPLEMENTARY DATA
- SUPPLEMENTARY DATA
- SUPPLEMENTARY DATA
- SUPPLEMENTARY DATA
- SUPPLEMENTARY DATA
- SUPPLEMENTARY DATA
- SUPPLEMENTARY DATA
