# Supplementary material for: Free energy landscape and transition pathways from Watson–Crick to Hoogsteen base pairing in free duplex DNA
Source: Nucleic Acids Res. 2015 Aug 6;43(16):7769–78. doi: 10.1093/nar/gkv796 (PMC4652778; doi:10.1093/nar/gkv796)
Supplement: SUPPLEMENTARY DATA [file supp_gkv796_nar-01190-f-2015-File008.docx]

**Supplementary data**

**Free energy landscape and transition pathways from Watson-Crick to Hoogsteen base pairing in free duplex DNA**

Changwon Yang^1^, Eunae Kim^2^, and Youngshang Pak^1*^

^1^ Department of Chemistry and Institute of Functional Materials, Pusan National University, Busan 609-735, S. Korea

^2^ College of Pharmacy, Chosun University, Gwangju 501-759, S. Korea

**
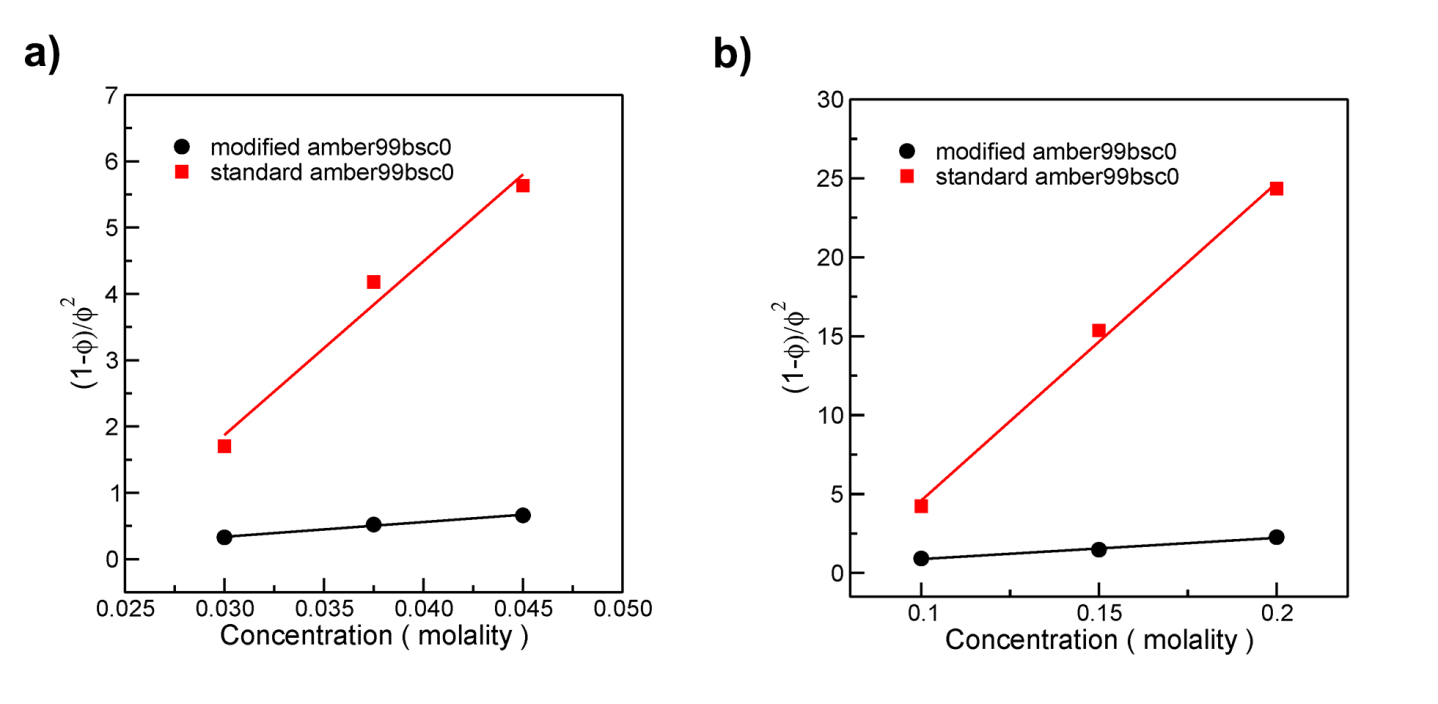
**

**Figure S1.** Aggregation simulation of deoxyribonucleoside solutions for 50 ns at 298 K and 1.0 atm. The *x*-axis is the initial molal concentration ($m_{T}$) of deoxyribonucleoside monomers in water and the y-axis is , where *φ* is the osmotic coefficient. (a) Aggregation simulation of deoxyadenosines (dAs) in water. The amber99bsc0 force field led to severe aggregation of dAs in water (*K_A_* = 262 M^-1^), but the modified amber99bsc0 yielded an estimated association constant of *K_A_* = 22 M^-1^ ($\Delta G_{A}^{0}$= -1.8 kcal/mol), which is then better agreement with a thermal osmometry experimental result (*K_A_* = 12 M^-1^;$\Delta G_{A}^{0}$= -1.5 kcal/mol) (1). (b) Aggregation simulation of deoxythymidines (dTs) in water. As expected, the standard amber99bsc0 led to a large association constant of *K_A_* ≈ 201 M^-1^, whereas the modified amber99bsc0 produced an improved association constant *K_A_* = 14 M^-1^ ($\Delta G_{A}^{0}$= -1.6 kcal/mol), which became closer to the experimental value of *K_A_* = 0.9 M^-1^ ($\Delta G_{A}^{0}$= 0.07 kcal/mol) (1).


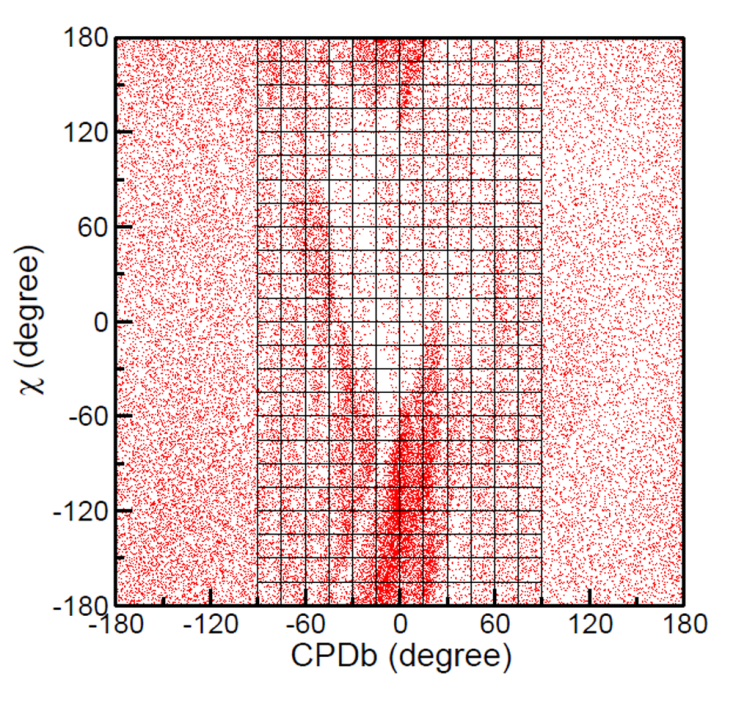


**Figure S2.** Two-dimensional (2D) scatter plot of (CPDb, χ) resulting from a well-tempered meta-dynamics trajectory of 50 ns (2). In this simulation, the upper and lower base pairs of the target A-T base pair were restrained to prevent unrealistic structural distortions of B-DNA. Each rectangular grid represents a window. Any point in each window was chosen as the initial state for the 2D-umbrella-MD simulation. In the well-tempered meta-dynamics scheme, the width of 2D-gaussian hill is 9.0 degrees and the hill height was initially set to 1.0 kcal/mol. The hill deposition interval is 1.0 ps and the bias factor is 15.


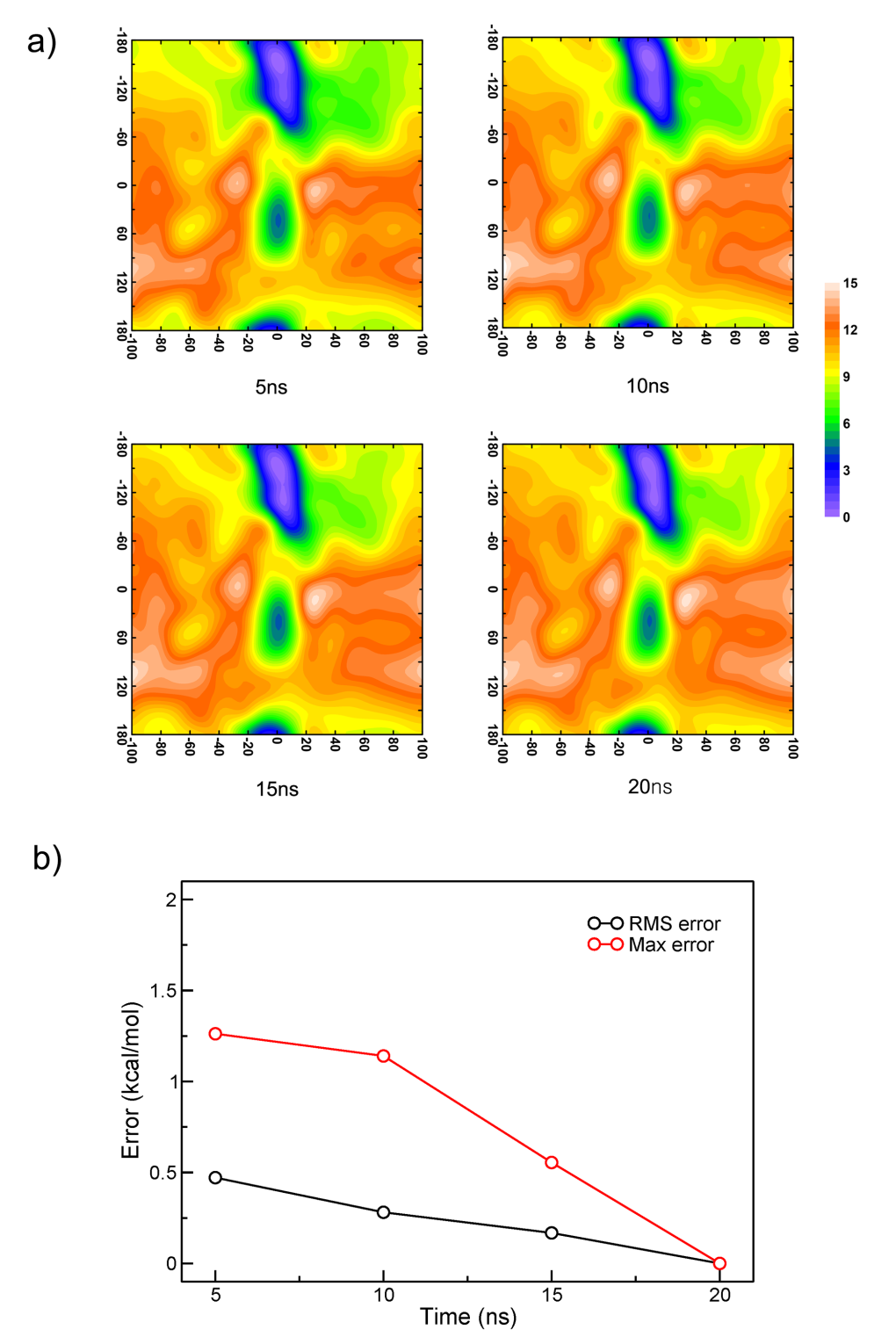


**Figure S3.** Convergence of the umbrella sampling simulation for 20 ns. (a) Cumulative free energy surfaces for simulation times of 5, 10, 15, and 20 ns. The X-axis is CPDb and the Y-axis is χ-angle. (b) Root mean square (RMS) and maximum absolute errors for the free energy surface obtained using the 20 ns trajectory. The error was calculated by $error_{RMS}(t)=\sqrt{\frac{1}{N\cdot M}\sum_{i=1}^{N} \sum_{j=1}^{M} \left(F(i,j;t)-F(i,j;t=20ns)\right)^{2}}$, where *N* and *M* are total numbers of grid points along *X* and *Y*, respectively, and *F* (*i*, *j* ; *t*) is the simulated free energy value at (*i*, *j*) grids in the cumulative time of *t*.


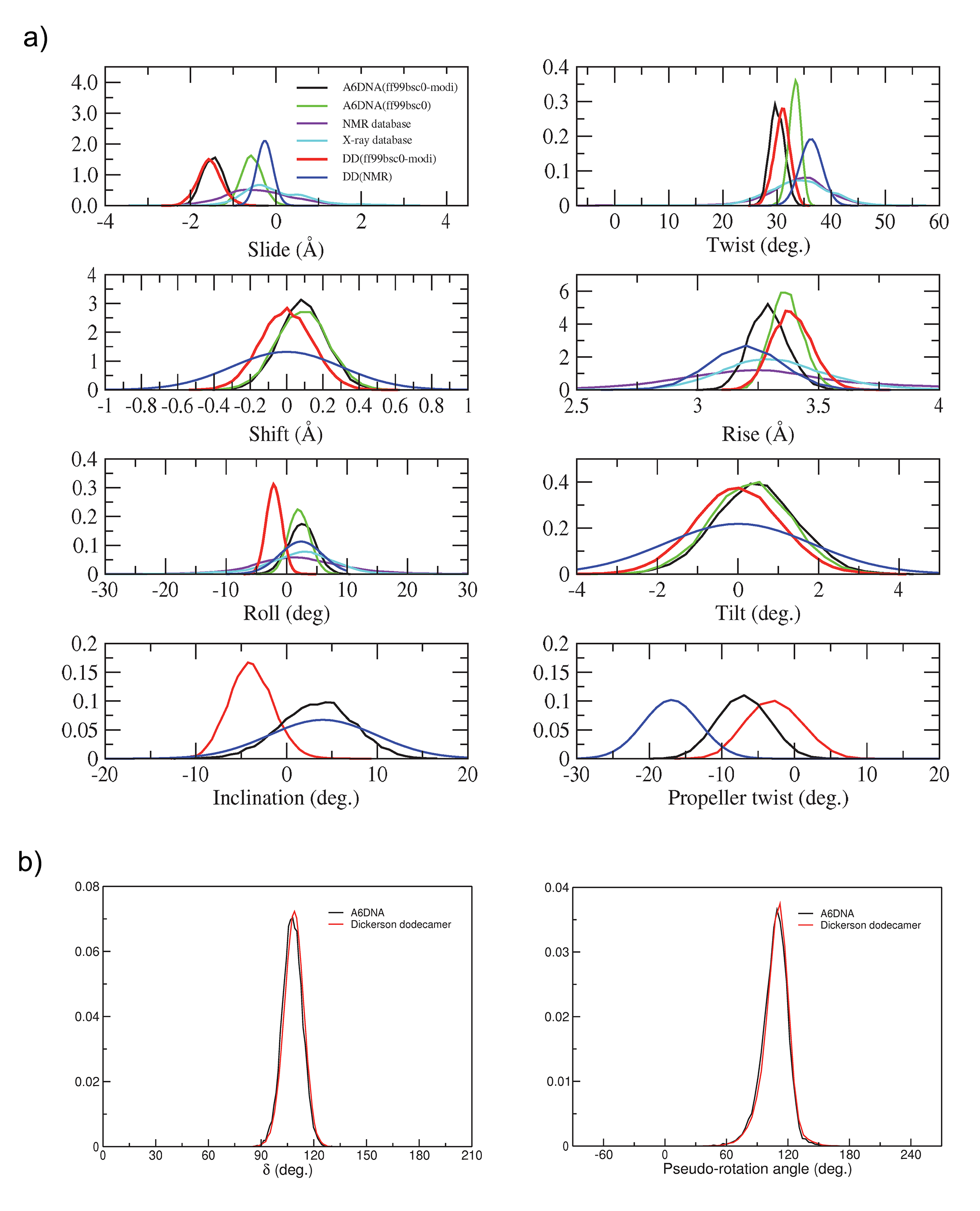


**Figure S4**. (a) Distributions of DNA base parameters of Slide, Twist, Roll, Rise, Shift, Tilt, Inclination and Propeller twist (3). MD simulations of A_6_-DNA at 300 K for 1 μs were performed using both modified amber99bsc0 (black) and original amber99bsc0 (green). In addition, a similar MD simulation was run for the B-form of Dickerson dodecamer (DD) using the modified amber99bsc0 (red). In comparison, the statistical distributions of base pair parameters extracted from NMR (violet) and X-ray (cyan) databases of B-form DNAs (4) and NMR structure of DD (blue, PDB entry: 1NAJ) (5) were included. Distributions of NMR structures of DD were obtained by estimating average values and standard deviations from the published five NMR structures of DD and assuming normal distribution. (b) Distributions of torsional delta-angle (C5’-C4’-C3’-O3’) and pseudo-rotation angle in sugar ring (6) for A_6_-DNA and DD. The distributions of both angles fall into those of typical B-form DNA: delta torsional angle of 70-180° and pseudo-rotation angle of 50-240° (7).


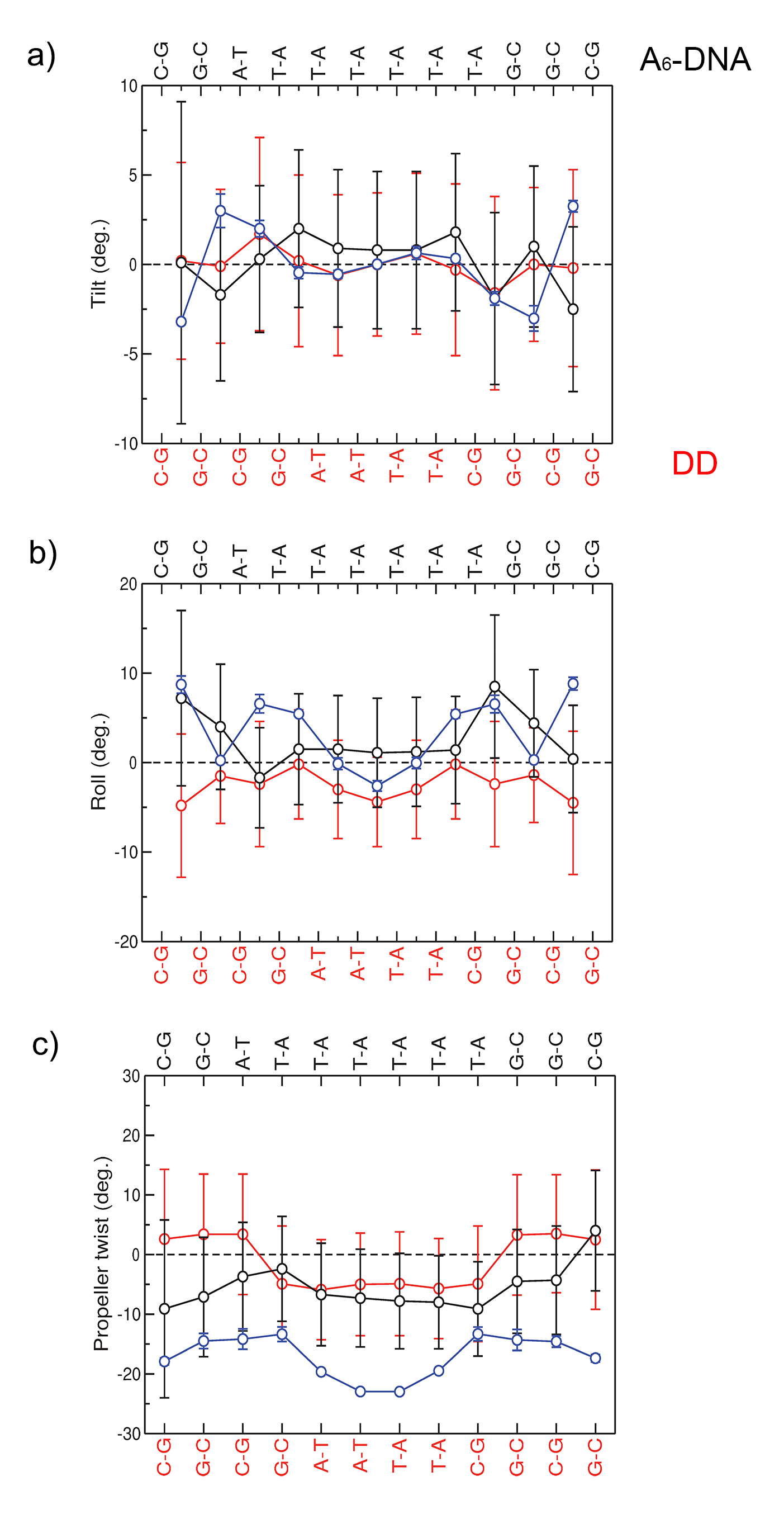


**Figure S5**. Base-pair position dependent (a) Tilt, (b) Roll, and (c) Propeller twist for A_6_-DNA (black), Dickerson dodecamer (DD) (red). In comparison, the same set of parameters obtained from the NMR structures of DD (DD, PDB entry: 1NAJ) are colored blue. These DNA parameters were averaged in 1 μs MD simulations at 300 K using the modified amber99bsc0 force field. The error bars represent a standard deviation.

**Movies in mp4 format for the six representative transition pathways**: **R1, R2, R3, L1, L2, and L3**. These representative movies were created by using a set of conformations sampled along the minimum free energy paths.

**References**

1. Solie, T.N. and Schellman, J.A. (1968) The interaction of nucleosides in aqueous solution. *J. Mol. Biol.*, **33**, 61-77.

2. Barducci, A., Bussi, G. and Parrinello, M. (2008) Well-tempered metadynamics: A smoothly converging and tunable free-energy method. *Phys. Rev. Lett.*, **100,** 020603.

3. Dickerson, R.E., Bansal,M., Calladine,C.R., Diekmann,S., Hunter,W.N., and Kennard, O., von Kitzing,E., Lavery,R., Nelson,H.C.M., Olson,W.K. et al. (1989) Definitions and Nomenclature of Nucleic-Acid Structure Parameters. *J. Mol. Biol.*, **205**, 787-791.

4. Gaillard, T. and Case, D.A. (2011) Evaluation of DNA Force Fields in Implicit Solvation. *J. Chem. Theory Comput.*, **7**, 3181-3198.

5. Wu, Z., Delaglio, F., Tjandra, N., Zhurkin, V.B. and Bax, A. (2003) Overall structure and sugar dynamics of a DNA dodecamer from homo- and heteronuclear dipolar couplings and 31P chemical shift anisotropy. *J. Biomol. NMR*, **26**, 297-315.

6. Altona, C. and Sundaral.M. (1972) Conformational-Analysis of Sugar Ring in Nucleosides and Nucleotides - New Description Using Concept of Pseudorotation. *J. Am. Chem. Soc.*, **94**, 8205.

7. Dickerson, R.E. and Ng, H.L. (2001) DNA structure from A to B. *Proc. Natl. Acad. Sci. U.S.A.*, **98**, 6986-6988.
